# Apatinib Plus Camrelizumab With/Without Chemoembolization for Hepatocellular Carcinoma: A Real-World Experience of a Single Center

**DOI:** 10.3389/fonc.2021.835889

**Published:** 2022-01-31

**Authors:** Shuguang Ju, Chen Zhou, Chongtu Yang, Chaoyang Wang, Jiacheng Liu, Yingliang Wang, Songjiang Huang, Tongqiang Li, Yang Chen, Yaowei Bai, Wei Yao, Bin Xiong

**Affiliations:** ^1^ Department of Radiology, Union Hospital, Tongji Medical College, Huazhong University of Science and Technology, Wuhan, China; ^2^ Hubei Province Key Laboratory of Molecular Imaging, Wuhan, China

**Keywords:** targeted therapy, immunotherapy, transarterial chemoembolization, hepatocellular carcinoma, comprehensive therapy

## Abstract

**Objective:**

This study was conducted in order to compare the efficacy and safety of transarterial chemoembolization (TACE) plus apatinib plus camrelizumab (TACE+AC) and apatinib plus camrelizumab (AC) in the treatment of unresectable hepatocellular carcinoma (HCC) in a real-world setting.

**Methods:**

In this single-center retrospective study, the data of patients with unresectable HCC who had received TACE+AC or AC treatment during March 2017 to May 2021 were assessed. Patients in the AC group received intravenous administration of camrelizumab 200 mg every 3 weeks and oral apatinib 250 mg/day treatment. Patients in the TACE+AC group received the same dose of camrelizumab and apatinib 1 week after TACE. The primary endpoint of the study was overall survival (OS), objective response rate (ORR), disease control rate (DCR), and adverse events (AEs) as the secondary endpoints.

**Results:**

A total of 108 patients were enrolled in the study. There were 52 patients in the AC group and 56 patients in TACE+AC group. Median OS was significantly longer in the TACE+AC group than in the AC group (24.8 vs. 13.1 months; *P* = 0.005). Patients in the TACE+AC group achieved a higher ORR [24 (42.9%) vs. 9 (17.3%), *P* = 0.004] than those in the AC group. Patients in the TACE+AC group also achieved a higher disease control rate (DCR) [48 (85.7%) vs. 30 (57.7%), *P* = 0.001] than patients in the AC group. There was no significant difference in the incidence of AEs related to apatinib and camrelizumab between the two groups, except for gastrointestinal reaction (*P* > 0.05, all; *P* < 0.05, gastrointestinal reaction).

**Conclusion:**

TACE plus apatinib plus camrelizumab significantly improved OS, ORR, and DCR over apatinib plus camrelizumab in patients with unresectable HCC. AEs were tolerable and manageable.

## Introduction

Liver cancer is the sixth most common cancer and the fourth leading cancer-related cause of death worldwide (1). Hepatocellular carcinoma (HCC) is the main histological subtype of liver cancer and accounts for about 90% of cases (2). Since the onset of HCC is insidious, most patients present with advanced stage and have a poor prognosis (3, 4).

Transcatheter therapy that included transarterial embolization (TAE) and transarterial chemoembolization (TACE) has been used for unresectable hepatic tumors (5, 6). Compared with TAE, TACE can increase the intertumor concentration of the chemotherapeutic agent. Doxorubicin is the preferred and most common chemotherapeutic agent used in TACE and it is considered an immunogenic drug, potentiating immunogenic cell death (6, 7). TACE is widely used in the treatment of intermediate and advanced HCC, especially unresectable hepatocellular carcinoma (8, 9). However, TACE leads to hypoxia in tumor tissue, resulting in upregulation of hypoxia inducible factor 1α (HIF-1α), which in turn induces the expression of vascular endothelial growth factor (VEGF) and increases tumor angiogenesis, thus promoting tumor growth and metastasis (10). Preclinical models have shown that the combination of anti-angiogenic therapy with TACE reduced tumor volume and vessel density and prolonged survival compared with TACE alone (11).

Apatinib is a novel oral small molecule VEGFR tyrosine kinase inhibitor, which can highly selectively inhibit the tyrosine kinase activity of VEGFR-2 and reduce tumor neoangiogenesis. A prospective, randomized, multicenter phase II clinical trial of apatinib for the first-line treatment of advanced HCC showed that apatinib improved patient survival and that adverse events were tolerable (12). Qiu et al. (13) suggested that TACE in combination with apatinib for unresectable HCC could improve the efficacy compared with TACE alone.

The identification of immune checkpoint molecules has provided a theoretical basis for the development of immunotherapy for HCC, such as programmed cell death-1 (PD-1) and programmed cell death-ligand 1 (PD-L1) (14). Tumor vascular abnormalities are the characteristic of most solid tumors and contribute to immune escape. Combining anti-angiogenic drugs with immunotherapy can improve the effectiveness of immunotherapy and improve patient prognosis (15). IMbrave150 showed that atezolizumab combined with bevacizumab had significantly better overall survival and progression-free survival than sorafenib in patients with unresectable HCC (3). The RESCUE trial also demonstrated the benefits of anti-angiogenic drugs in combination with immunotherapy in advanced HCC (16). Camrelizumab in combination with apatinib has a safety and efficacy profile, with objective response rate (ORR) rates of 34.3% and 22.5% for first-line and second-line treatment, respectively (16). More than half of the patients in both the IMbrave150 trial and the RESCUE trial had received local or interventional therapy (3, 16). Due to the fact that TACE can induce immunogenic tumor cell death, TACE combined with immunotherapy can enhance the efficacy (7, 17).

Apatinib in combination with camrelizumab improves clinical benefit in patients with advanced HCC, whereas TACE combined with apatinib plus camrelizumab for unresectable HCC patients has not yet been reported (16). We conducted this real-world study to explore the safety and efficacy of TACE in combination with camrelizumab plus apatinib compared with camrelizumab plus apatinib alone in patients with unresectable HCC.

## Materials and Methods

### Patients

The data analysis for this study was based on real-world data. This retrospective study was approved by the Institutional Ethics Review Board of Wuhan Union Hospital and was conducted in accordance with the Declaration of Helsinki. Written informed consent was waived, as this study was retrospective. From March 2017 to May 2021, a total of 153 HCC patients who underwent apatinib (Jiangsu Hengrui Medicine Co., Ltd., Jiangsu, China) plus camrelizumab (Jiangsu Hengrui Medicine Co., Ltd., Jiangsu, China) (AC) treatment were screened at our hospital. These patients were classified into TACE + apatinib + camrelizumab (TACE+AC) group and apatinib + camrelizumab (AC) group according to whether they had received TACE treatment or not. Patients in the TACE+AC group were from the departments of intervention, oncology and surgery, while patients in the AC group were from the departments of oncology and surgery. The main inclusion criteria were as follows: 1) diagnosed as unresectable HCC based on imaging techniques and/or biopsy according to the American Association for the Study of the Liver Diseases (AASLD) (18); 2) at least one measurable target lesion, including multinodular HCC; 3) Barcelona Clinic Liver Cancer (BCLC) tumor stage B or C; 4) Child–Pugh stage A or B7; 5) normal renal function, normal coagulation, or correction by appropriate treatment; 6) Eastern Cooperative Oncology Group Performance Status (ECOG PS) 0 or 1 within 1 week before treatment; and 7) the TACE+AC group received at least one cycle of TACE combined with apatinib plus camrelizumab and the AC group received at least one dose of apatinib–camrelizumab. The exclusion criteria included the following: 1) diffuse HCC or tumor burden exceeding 70% of the whole liver; 2) complete obstruction of the main portal vein by tumor thrombi; 3) ECOG PS >1; 4) Child–Pugh stage C; 5) severe coagulation disorders, renal dysfunction, and cardiopulmonary dysfunction which cannot be corrected; and 6) incomplete data.

### Treatment Protocol

The TACE procedure was performed as described by Wang et al. (19) Patients are treated with TACE under local anesthesia by interventional radiologists with over 10 years of experience. Proactive measures were taken to minimize the amount of absorbed radiant dose to the operators and patients during the operation (20). The tip of a 5-French (F) catheter (COOK) was inserted into the celiac trunk artery and superior mesenteric artery sequentially for high-pressure angiograph through the femoral artery so that the tumor feed artery could be identified. Then a 2.7-F microcatheter (Terumo, Tokyo, Japan) was super-selected into the tumor feed artery for embolization. The embolization modality was determined by the interventional physicians and patients, including the following: 1) different diameter drug-eluting bead (Jiangsu Hengrui Medicine Co., Ltd., Jiangsu, China) loaded with 60 mg doxorubicin (DOX); and 2) the iodine oil–DOX emulsion, a water-in-oil type of chemoembolization, which was prepared by using doxorubicin mixed with lipiodol (21). For drug-eluting bead (DEB)-TACE, the operator slowly injected the drug-eluting bead through the microcatheter until the tumor staining disappeared. For conventional TACE (cTACE), the operator slowly injected iodine oil–DOX emulsion through the microcatheter, followed by embolization of the vascular trunk with absorbable gelatin sponge particles until the tumor staining disappeared. The TACE process was repeated when the tumor still had a blood supply on review using dynamic CT or MRI and a Child–Pugh classification of A or B. Two experienced radiologists evaluated the CT or MRI images of the patient to assess whether the tumor still had an arterial blood supply (22).

Patients in the AC group received intravenous administration of camrelizumab 200 mg every 3 weeks and oral apatinib 250 mg/day treatment. Patients in the TACE+AC group received the same dose of camrelizumab and apatinib 1 week after TACE. Apatinib was suspended 3 days before the following TACE procedure. The dose of camrelizumab and apatinib in patients who experienced adverse events (AEs) due to those agents was reduced, suspended, or discontinued.

### Study Endpoints

The primary endpoint was overall survival (OS). OS was defined from the date of the first TACE therapy to the date of death arising from any cause or the date of last contact in the TACE+AC group. In the AC group, OS was defined from date of the first combination therapy to the date of death arising from any cause or the date of last contact. Secondary endpoints of this study included ORR, disease control rate (DCR), and AEs. Tumor response was evaluated by two experienced radiologists using the modified Response Evaluation Criteria in Solid Tumors (mRECIST, version 1.1), including complete response (CR), partial response (PR), stable disease (SD), and progressive disease (PD). ORR was defined as CR+PR, and DCR was defined as CR+PR+SD. AEs were assessed based on the Common Terminology Criteria for Adverse Events (CTCAE, version 4.03).

### Statistical Methods

All data were analyzed using SPSS 21.0 statistical software (IBM Corp., Armonk, NY, USA). All the continuous variables were reported as means ± standard deviation (SD) or median (minimum, maximum), and categorical variables were expressed as numbers (percentages). Continuous data were compared using Student’s *t*-test, and categorical data were compared using the chi-squared or Fisher’s exact test. Survival curve was estimated by the Kaplan–Meier method, from which median OS was calculated, followed by comparison using the log-rank test. Factors affecting OS were predicted by multifactorial Cox regression analysis. A two-sided *P*-value <0.05 was considered significant.

## Results

### Patient Demographics

**Figure 1:**
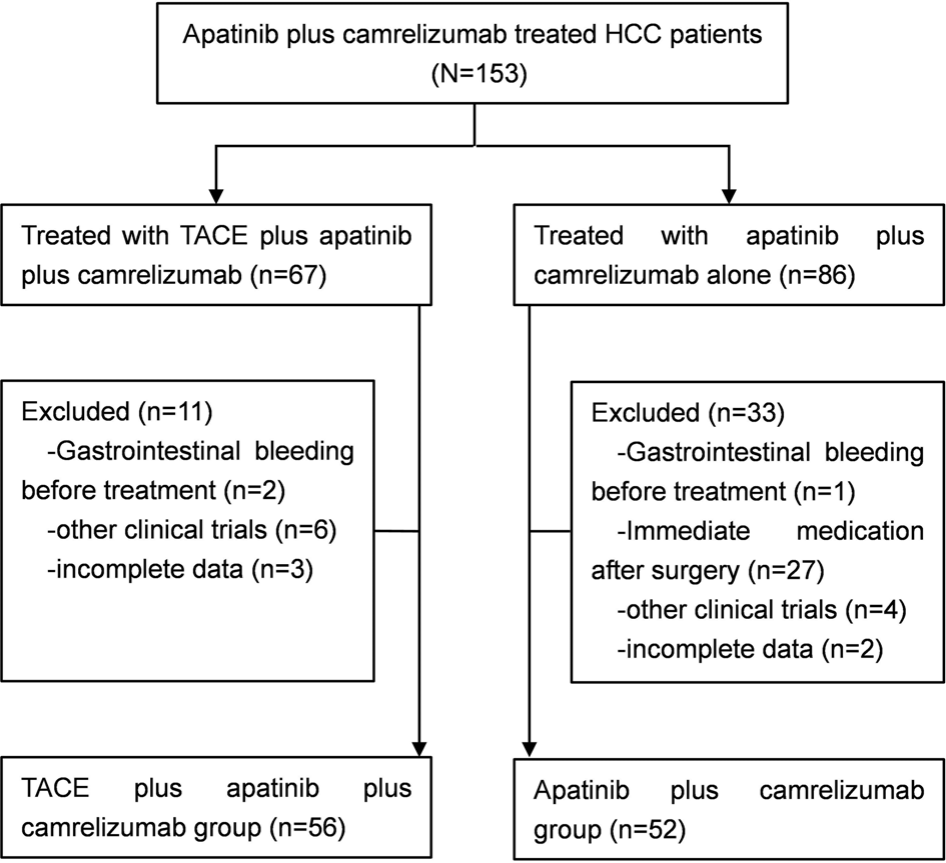
Study flow. HCC, hepatocellular carcinoma; TACE, transcatheter arterial chemoembolization.

Between March 2017 and May 2021, a total of 108 patients with unresectable HCC were enrolled in this study. Fifty-six patients underwent TACE combined with apatinib plus camrelizumab treatment in the TACE+AC group, while 52 patients underwent apatinib plus camrelizumab alone in the AC group (**Figure 1**). The two groups were comparable in terms of age, age group, sex, ECOG PS, Child–Pugh class, BCLC stage, liver cirrhosis, etiology, tumor distribution, tumor size, and laboratory parameters. The baseline characteristics were well balanced among the treatment groups, as shown in **Table 1**. The follow-up ended on October 1, 2021. Median follow-up time was 13.5 months.

**Table 1:** Patient characteristics at baseline.

Characteristics	AC group (*n* = 52)	TACE+AC group (*n* = 56)	*P*-value
Age (years), median (range)	55 (25–79)	52 (26–75)	0.978
Age group (years) (%)			0.949
<65	43 (82.7)	45 (80.4)	
≥65	9 (17.3)	11 (19.6)	
Sex (%)			0.931
Male	44 (84.6)	46 (82.1)	
Female	8 (15.4)	10 (17.9)	
ECOG PS (%)			0.673
0	22 (42.3)	27 (48.2)	
1	30 (57.7)	29 (51.8)	
Child–Pugh class (%)			0.979
A	41 (78.8)	43 (76.8)	
B	11 (21.2)	13 (23.2)	
BCLC stage (%)			0.102
B	5 (9.6)	13 (23.2)	
C	47 (90.4)	43 (76.8)	
Liver cirrhosis (%)			1.000
No	6 (11.5)	6 (10.7)	
Yes	46 (88.5)	50 (89.3)	
Etiology (%)			0.133
Hepatitis B	44 (84.6)	48 (85.7)	
Hepatitis C	6 (11.5)	2 (3.6)	
Non-B, non-C	2 (3.8)	6 (10.7)	
Tumor distribution (%)			0.912
Single	7 (13.5)	9 (16.1)	
Multiple	45 (86.5)	47 (83.9)	
Tumor size (cm, mean ± SD) (%)	8.6 ± 4.4	9.7 ± 4.9	0.254
<10 cm	31 (59.6)	31 (55.4)	0.801
≥10 cm	21 (40.4)	25 (44.6)	
Laboratory parameters			
RBC (10^9^/L, mean ± SD)	4.20 ± 0.71	4.11 ± 0.80	0.545
Hb (g/L, mean ± SD)	126.50 ± 16.15	124.02 ± 21.31	0.499
Platelet (10^9^/L, mean ± SD)	191.51 ± 84.90	191.35 ± 91.53	0.992
WBC (10^12^/L, mean ± SD)	5.63 ± 2.07	5.81 ± 1.97	0.640
Neutrophils (10^9^/L, mean ± SD)	3.77 ± 1.69	3.91 ± 1.75	0.659
Lymphocyte (10^9^/L)	1.22 ± 0.62	1.25 ± 0.44	0.740
NLR (mean ± SD)	3.87 ± 2.80	3.58 ± 2.52	0.564
ALT (U/L, mean ± SD)	49.57 ± 45.75	46.19 ± 25.58	0.633
AST (U/L, mean ± SD)	73.07 ± 56.32	67.62 ± 63.45	0.639
TBIL (μmol/L, mean ± SD)	21.24 ± 13.57	18.08 ± 12.00	0.201
ALP (U/L, mean ± SD)	182.24 ± 102.94	172.33 ± 102.96	0.618
TBA (μmol/L, mean ± SD)	19.71 ± 20.87	14.03 ± 20.85	0.160
ALB (g/L, mean ± SD)	37.80 ± 4.55	37.04 ± 4.60	0.391
AFP (ng/ml) (%)			0.913
<200	21 (40.4)	21 (37.5)	
≥200	31 (59.6)	35 (62.5)	

Data are median (range) or N (%).

TACE, transcatheter arterial chemoembolization; AC, apatinib plus camrelizumab; TACE+AC, TACE plus apatinib plus camrelizumab; ECOG PS, Eastern Cooperative Oncology Group Performance Status; BCLC, Barcelona Clinic Liver Cancer; RBC, red blood cell; Hb, hemoglobin; WBC, white blood cell; NLR, neutrophils/lymphocyte; ALT, alanine aminotransferase; AST, aspartate aminotransferase; TBIL, total bilirubin; ALP, alkaline phosphatase; TBA, total bile acid; ALB, albumin; AFP, alpha-fetoprotein.

### Efficacy

**Figure 2:**
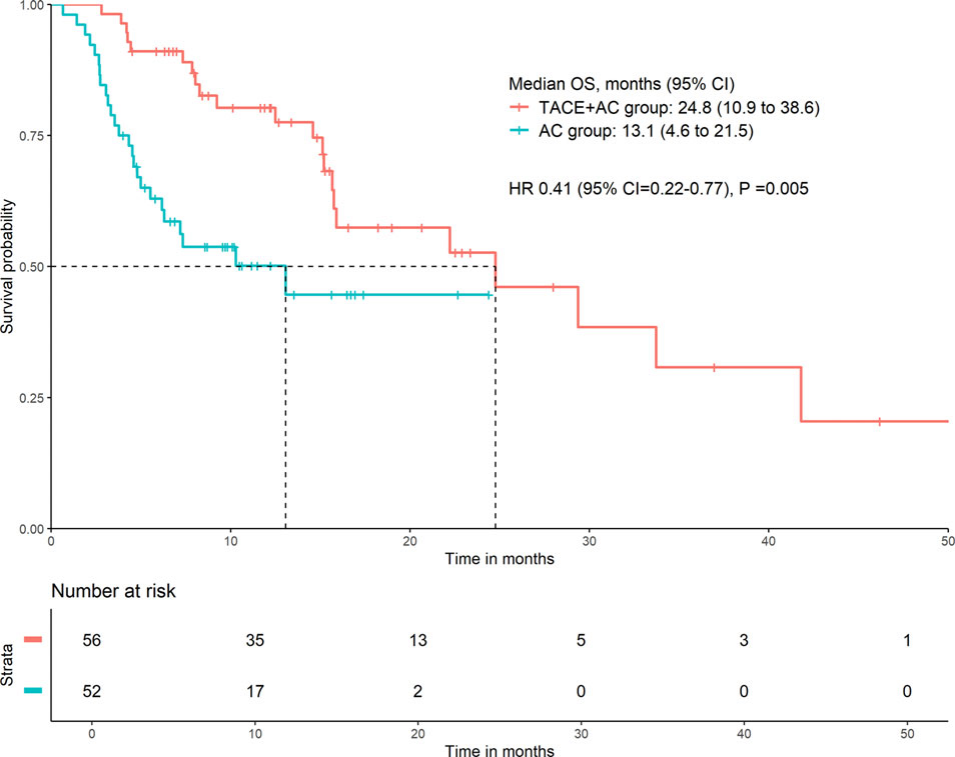
Kaplan–Meier curve for OS. OS, overall survival; HR, hazard ratio; CI, confidence interval; TACE+AC, TACE plus apatinib plus camrelizumab; AC, apatinib plus camrelizumab; TACE, transcatheter arterial chemoembolization.

**Figure 3:**
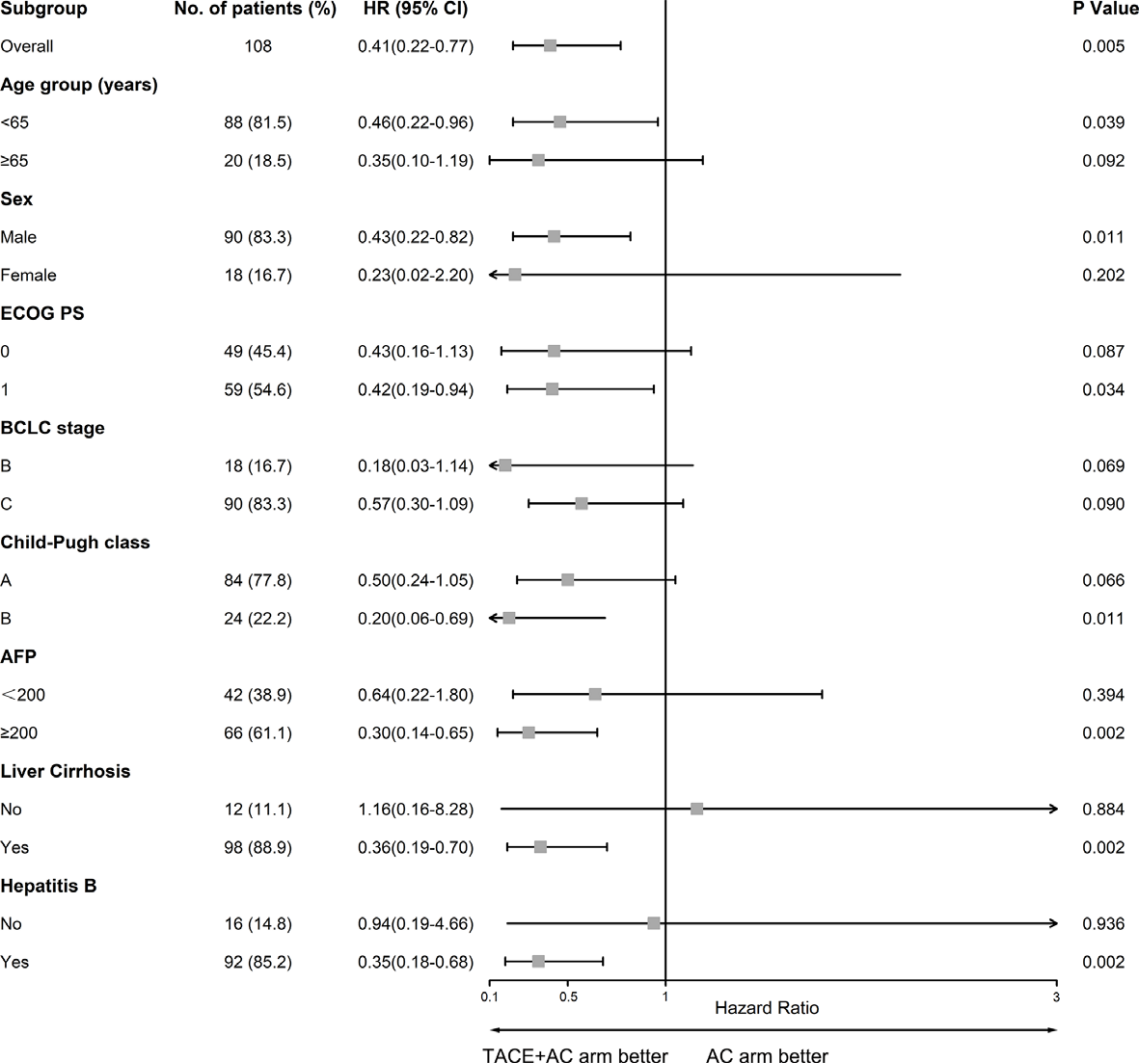
Forest plot of OS in subgroups of patients treated with TACE plus apatinib plus camrelizumab and apatinib plus camrelizumab. TACE, transcatheter arterial chemoembolization; ECOG PS, Eastern Cooperative Oncology Group Performance Status; BCLC, Barcelona Clinic Liver Cancer; AFP, alpha-fetoprotein.

Patients in the TACE+AC group had a median OS of 24.8 months (95% CI = 10.9–38.6) compared with 13.1 months (95% CI = 4.6–21.5) for those in the AC group (HR 0.41, 95% CI = 0.22–0.77, *P* = 0.005; **Figure 2**). Multivariable Cox regression showed that the independent risk factors for OS were group (HR = 0.314, 95% CI = 0.154–0.641, *P* < 0.001), age group (HR = 2.630, 95% CI = 1.326–5.216, *P* = 0.006), lymphocyte (HR = 0.388, 95% CI = 0.210–0.716, *P* = 0.002), and ALB (HR = 0.930, 95% CI = 0.866–0.998, *P* = 0.043) (**Table 2**). The subgroup analyses of OS are presented in **Figure 3**. TACE+AC provided a clinical benefit for OS in these subgroups as follows: age less than 65 years, ECOG PS score of 1, Child–Pugh classification of B, AFP greater than or equal to 200 ng/ml, male, liver cirrhosis, and hepatitis B infected.

**Table 2:** Univariate Cox proportional hazards regression model analysis for OS.

	Univariate analysis			Multivariate analysis		
	HR	95% CI	*P*-value	HR	95% CI	*P*-value
Group (TACE+AC group vs. AC group)	0.413	0.223–0.766	0.005	0.314	0.154–0.641	0.001
Sex (female vs. male)	0.738	0.312–1.744	0.488			
Age group (≥65 vs. <65)	2.319	1.235–4.357	0.009	2.630	1.326–5.216	0.006
ECOG PS (per 1 point increase)	1.723	0.935–3.174	0.081	–		
BCLC stage (C vs. B)	2.532	0.984–6.515	0.054	–		
Tumor size (per 1 point increase)	1.003	0.997–1.009	0.366			
Tumor distribution (multiple vs. single)	1.586	0.624–4.029	0.332			
Child–Pugh class (B vs. A)	1.980	1.049–3.738	0.035	–		
Liver cirrhosis (yes vs. no)	0.941	0.334–2.655	0.909			
AFP (≥200 vs. <200)	1.384	0.754–2.542	0.294			
RBC	0.999	0.685–1.456	0.994			
Hb	0.908	0.778–1.060	0.222			
Platelet	1.000	0.997–1.004	0.813			
WBC	0.999	0.984–1.015	0.928			
Neutrophils	0.984	0.828–1.168	0.851			
Lymphocyte	0.365	0.193–0.690	0.002	0.388	0.210-0.716	0.002
NLR	1.100	0.997–1.214	0.058	–		
ALT	0.998	0.988–1.008	0.677			
AST	1.003	0.998–1.007	0.233			
TBIL	1.015	0.996–1.035	0.124			
ALP	1.003	1.001–1.006	0.014	–		
TBA	1.009	0.999–1.019	0.065	–		
ALB	0.935	0.879–0.994	0.032	0.930	0.866–0.998	0.043

AC, apatinib plus camrelizumab; TACE+AC, TACE plus apatinib plus camrelizumab; OS, overall survival; HR, hazard ratio; CI, confidence interval; ECOG PS, Eastern Cooperative Oncology Group Performance Status; RBC, red blood cell; Hb, hemoglobin; WBC, white blood cell; NLR, neutrophils/lymphocyte; ALT, alanine aminotransferase; AST, aspartate aminotransferase; TBIL, total bilirubin; ALP, alkaline phosphatase; TBA, total bile acid; ALB, albumin; AFP, alpha-fetoprotein; TACE, transcatheter arterial chemoembolization.

The tumor responses of the two groups of patients are shown in **Table 3**. On the basis of mRECIST 1.1, there was a significant difference between the two groups of patients in terms of tumor response (*P* = 0.003). Patients in the TACE+AC group achieved a higher ORR [24 (42.9%) vs. 9 (17.3%), *P* = 0.004] than those in the AC group. Patients in the TACE+AC group also achieved a higher DCR [48 (85.7%) vs. 30 (57.7%), *P* = 0.001] than patients in the AC group.

**Table 3:** Summary of tumor response based on mRECIST 1.1.

	AC group (*n* = 52, %)	TACE+AC group (*n* = 56, %)	*P*-value	Overall (*n* = 108, %)
Tumor response			0.003	
CR	1 (1.9)	1 (1.8)		2 (1.9)
PR	8 (15.4)	23 (41.1)		31 (28.7)
SD	21 (40.4)	24 (42.9)		45 (41.6)
PD	22 (42.3)	8 (14.3)		30 (27.8)
ORR (CR+PR)	9 (17.3)	24 (42.9)	0.004	33 (30.6)
DCR (CR+PR+SD)	30 (57.7)	48 (85.7)	0.001	78 (72.2)

mRECIST, modified Response Evaluation Criteria in Solid Tumors; AC, apatinib plus camrelizumab; TACE+AC, TACE plus apatinib plus camrelizumab; TACE, transcatheter arterial chemoembolization; CR, complete response; PR, partial response; SD, stable disease; PD, progressive disease; ORR, objective response rate; DCR, disease control rate.

### Safety

No treatment-related deaths were observed (**Table 4**). The most common AEs associated with apatinib and camrelizumab observed in the AC group were hand-foot skin reaction (40.4%), hypertension (32.7%), proteinuria (26.9%), abnormal thyroid function [hypothyroidism (19.2%) and hyperthyroidism (5.8%)], and gastrointestinal reaction (defined as diarrhea and abdominal distension) (17.3%). Typical AEs related to TACE included fever (53.6%), nausea and vomiting (21.4%), pain (42.9%), and gastrointestinal reaction (33.9%). Because TACE treatment was available in the TACE+AC group, there was no significant difference in the incidence of AEs related to apatinib and camrelizumab between the two groups, except for gastrointestinal reaction (*P* > 0.05, all; *P* < 0.05, gastrointestinal reaction). One patient in the TACE+AC group suffered liver abscess and recovered after tube drainage and anti-infection treatment. According to CTCAE 4.03, most AEs were classified as grades 1–2, including 5 patients who experienced mouth ulcers [2 (3.8%] in the AC group, 3 (5.4%) in the TACE+AC group, *P* > 0.05], 1 patient who developed hepatic encephalopathy in the TACE+AC group, 7 patients who had rash [3 (5.8%) in the AC group, 4 (7.1%) in the TACE+AC group, *P* > 0.05], 14 patients who had reactive cutaneous capillary endothelial proliferation (RCCEP) [8 (15.4%) in the AC group, 6 (10.7%) in the TACE+AC group, *P* > 0.05], 3 patients who were observed to have myocarditis [1 (1.9%) in the AC group, 2 (3.6%) in the TACE+AC group, *P* > 0.05], and 5 patients who had hemorrhage and upper gastrointestinal tract [2 (3.8%) in the AC group, 3 (5.4%) in the TACE+AC group, *P* > 0.05]. One patient in the AC group developed grade 3 immune myocarditis and then discontinued the subsequent use of camrelizumab. All patients were corrected after symptomatic treatment.

**Table 4:** Treatment-related adverse events.

*n* (%)	AC group (*n* = 52)		TACE+AC group (*n* = 56)	
	All grade	Grade ≥3	All grade	Grade ≥3
Fever	2 (3.8)	0 (0)	30 (53.6)	1 (1.8)
Nausea and vomiting	0 (0)	0 (0)	12 (21.4)	0 (0)
Pain	0 (0)	0 (0)	24 (42.9)	5 (8.9)
Liver abscess	0 (0)	0 (0)	1 (1.8)	1 (1.8)
Hand-foot skin reaction	21 (40.4)	2 (3.8)	21 (37.5)	2 (3.6)
Hypertension	17 (32.7)	1 (1.9)	17 (30.4)	0 (0)
Fatigue	11 (21.2)	0 (0)	13 (23.2)	0 (0)
Mouth ulcers	2 (3.8)	0 (0)	3 (5.4)	0 (0)
Proteinuria	14 (26.9)	0 (0)	14 (25.0)	0 (0)
Hepatic encephalopathy	0 (0)	0 (0)	1 (1.8)	0 (0)
Rash	3 (5.8)	0 (0)	4 (7.1)	1 (1.8)
Hypothyroidism	10 (19.2)	0 (0)	8 (14.3)	0 (0)
Hyperthyroidism	3 (5.8)	0 (0)	4 (7.1)	0 (0)
RCCEP	8 (15.4)	0 (0)	6 (10.7)	0 (0)
Gastrointestinal reaction	9 (17.3)	5 (9.6)	19 (33.9)	2 (3.6)
Myocarditis	1 (1.9)	1 (1.9)	2 (3.6)	0 (0)
Hemorrhage, upper GI	2 (3.8)	0 (0)	3 (5.4%)	0 (0)

Gastrointestinal reaction, defined as diarrhea and abdominal distension.

AC, apatinib plus camrelizumab; TACE+AC, TACE plus apatinib plus camrelizumab; TACE, transcatheter arterial chemoembolization; RCCEP, reactive cutaneous capillary endothelial proliferation; GI, gastrointestinal tract.

## Discussion

Treatment options for unresectable HCC have rapidly evolved in recent years. Treatment options including TACE, multikinase inhibitors, and immune checkpoint inhibitors have been applied to patients with unresectable HCC (8). However, the efficacy of monotherapy still needs to be improved, and combination therapy could provide additional clinical benefit to patients (3, 4, 15, 23). The IMbrave150 trial showed that atezolizumab plus bevacizumab prolongs progression-free survival (PFS) more than sorafenib patients with unresectable HCC, the median PFS in the atezolizumab plus bevacizumab group was 6.8 months, and in the sorafenib group, it was 4.3 months. The hazard ratio of death for atezumab–bevacizumab was 0.58 (95% CI = 0.42–0.79) compared with sorafenib (3). The RESCUE trial observed that PFS was 5.7 and 5.5 months for first- and second-line treatment of unresectable HCC with apatinib in combination with camrelizumab, respectively (16). In the IMbrave150 trial, 48% of patients in the atezumab–bevacizumab group had received prior local therapy, while more than 60% of patients in the RESCUE trial had received prior interventional therapy (3, 16).

This real-world retrospective study showed significantly better overall survival and tumor response rate outcomes with TACE plus apatinib plus camrelizumab than with apatinib plus camrelizumab in patients with unresectable HCC. Cao et al. (24) reported TACE combined with lenvatinib and sintilimab for unresectable HCC with a median OS of 23.6 months and ORR of 46.7%, which is similar to our results. These suggest that TACE combined with targeted therapy plus immunotherapy is a promising treatment option in unresectable HCC. One possible reason is that TACE can reduce tumor burden by inducing tumor necrosis through cytotoxic effect and tumor tissue ischemia (25). Another possible reason is the use of doxorubicin in the TACE+AC group during TACE treatment. Doxorubicin, a chemotherapeutic reagent widely used in TACE treatment, could induce and potentiate immunogenic cell death (7). Third, tumor tissue necrosis after TACE treatment causes tumor tissue to release tumor antigens and enables increased expression of PD-1 and PD-L1, improving tumor recognizability (26). At the same time, TACE also induces immunogenic cell death, which could stimulate an immune response in the peripheral system that may be further amplified by immune checkpoint blockade (7).

In this study, the ORR according to mRECIST 1.1 was significantly higher in patients in the TACE+AC group than in the AC group (42.9% vs. 17.3%). The ORR of the TACE+AC group was comparable to the results of the RESCUE trial (42.9% vs. 45.7%), but the ORR of the AC group was lower than that of the RESCUE trial (17.3% vs. 45.7%) (16). The possible reasons were that a higher percentage of patients in the AC group had a Child–Pugh classification of B compared with the RESCUE study (21.2% vs. 0%), and none of the patients in the AC group had been treated with interventional therapy (0% vs. 62.9%). The ORR in the TACE+AC was comparable to the RESCUE study probably because 23.2% of the patients had poor liver function levels. This might indirectly demonstrate the advantage of TACE combined with apatinib plus camrelizumab. The ORR of the TACE+AC group was better than or comparable to the results of the IMbrave150 trial (atezolizumab plus bevacizumab: ORR = 33.2%), the phase 1b KEYNOTE-524 trial (lenvatinib plus pembrolizumab: ORR = 46.0%), and the ORIENT-32 trial (sintilimab plus bevacizumab: ORR = 24%), whereas the ORR for the AC group was lower than the results of those studies (3, 4, 27). It also might be attributable to the difference in those study populations. Compared with the AC group, the IMbrave150 trial had a higher proportion of patients who had received local therapy (48%) and a lower proportion of patients with hepatitis B (49%) and poor liver function (Child–Pugh B = 0%), the KEYNOTE-524 trial had a lower percentage of patients with hepatitis B (19%) and poor liver function (Child–Pugh B = 2%), and 66% of patients in the ORIENT-32 trial had received TACE. Poor liver function and hepatitis B-associated HCC might have a poor prognosis after tumor treatment (27). We also argued that TACE improved the clinical benefit of these patients possibly.

Subanalyses of OS were performed based on various factors. TACE+AC provided a higher OS in some subgroup analyses. Other subgroup analyses including patients older than 65 years (18.5%), female (16.7%), without liver cirrhosis (11.1%), and without hepatitis B infection (14.8%) had wide 95% CI ranges, which we considered might be caused by the small sample size. By Cox multivariate regression analysis, we found that group, age group, lymphocyte absolute value, and albumin level were independent risk factors affecting the prognosis of patients in this study.

This study suggested that both TACE+AC and AC were tolerable and had a well-managed safety profile. Since postembolization syndrome (fever, nausea and vomiting, and pain) and liver abscess were likely associated with TACE, the incidence of both of the TACE+AC group was higher than that of the AC group. Hand-foot skin reaction, hypertension, fatigue, mouth ulcers, proteinuria, hepatic encephalopathy, and rash were likely associated with aptinib, while the occurrence of abnormal thyroid function, RCCEP, and myocarditis were likely related with camrelizumab, and the incidence of these AEs was generally consistent between the two groups. Gastrointestinal reaction was thought to be possibly caused by the combination therapy, with a higher incidence in the TACE+AC group. No new or unexpected toxic effects happened (16, 28).

Upper gastrointestinal bleeding may occur in HCC patients in combination with chronic hepatitis B cirrhosis. A total of 85.2% of patients had hepatitis B infection and 88.9% had cirrhosis in this study. This study showed that the incidence of upper gastrointestinal tract hemorrhage was 4.6% (5/108), consisting of 2 (3.8%) patients in the AC group and 3 (5.4%) patients in the TACE+AC group, which was similar to the results in the RESCUE study, and none was considered treatment related (16). One patient developed hepatic encephalopathy, which may be related to the damage of the blood–brain barrier due to the damage of brain endothelial cells by apatinib (28). In addition, for TACE-based combination therapy, it is necessary that interventional radiological procedures are performed by experienced radiologists to reduce stochastic and non-stochastic risks from X-ray exposure and to minimize the increase of X-ray-induced complications (20). Although these patients recovered after treatment, we still need to delve into the specific causes of these AEs and screen the population suitable for this treatment option.

Tovoli et al. (29) reported that better overall survival was associated with longer treatment cycles and less permanent discontinuation due to toxicity. Moreover, the occurrence of adverse events can also predict patient prognosis. Granito et al. (30) showed that post-treatment transaminase elevation predicted the objective response to TACE. The results regarding the management of adverse events with TACE+AC and its relationship with prognosis will be reported in our subsequent study.

There were several limitations in this study. First, this study was a single-center retrospective study with a small population, which might reduce the statistical power of the study. Second, some patients in the TACE+AC group did not receive apatinib plus camrelizumab therapy after their first TACE treatment because of the financial difficulties of their families and the high price of the drugs. If they had received combination therapy earlier, they might have achieved a higher clinical benefit. Third, in TACE+AC group, the TACE treatments included cTACE and DEB-TACE, with a consequent bias of different treatments. Finally, the follow-up time was relatively short and endpoint events had not been observed in some patients. Therefore, further prospective studies or randomized controlled trials with larger sample sizes are needed to validate these results in the future.

In summary, this study showed that TACE plus apatinib plus camrelizumab demonstrated superior efficacy to apatinib plus camrelizumab for patients with unresectable HCC. Both treatments were safe and well tolerated. It provides a strong guidance for the treatment of intermediate and advanced HCC.

## Data Availability Statement

The original contributions presented in the study are included in the article/supplementary material. Further inquiries can be directed to the corresponding author.

## Ethics Statement

The studies involving human participants were reviewed and approved by Wuhan Union Hospital. Written informed consent for participation was not required for this study in accordance with the national legislation and the institutional requirements.

## Author Contributions

Planning and conducting the study: BX and SJ. Collecting the data: SJ, CY, YW, WY, CZ, YB, CW, and SH. Interpreting the data: YB, JL, CY, YC, YW, and TL. Drafting the manuscript: SJ, CZ, and CY. Revising the manuscript: BX. All authors contributed to the article and approved the submitted version.

## Funding

This work was funded by grants from the National Natural Science Foundation of China (81873917).

## Conflict of Interest

The authors declare that the research was conducted in the absence of any commercial or financial relationships that could be construed as a potential conflict of interest.

## Publisher’s Note

All claims expressed in this article are solely those of the authors and do not necessarily represent those of their affiliated organizations, or those of the publisher, the editors and the reviewers. Any product that may be evaluated in this article, or claim that may be made by its manufacturer, is not guaranteed or endorsed by the publisher.
